# ANAT 2.0: reconstructing functional protein subnetworks

**DOI:** 10.1186/s12859-017-1932-1

**Published:** 2017-11-16

**Authors:** Yomtov Almozlino, Nir Atias, Dana Silverbush, Roded Sharan

**Affiliations:** School of Computer Science, Tel Aviv University, 69978 Tel Aviv, Israel

**Keywords:** Network inference, Protein-protein interaction network, Subnetwork reconstruction, Cytoscape plugin

## Abstract

**Background:**

ANAT is a graphical, Cytoscape-based tool for the inference of protein networks that underlie a process of interest. The ANAT tool allows the user to perform network reconstruction under several scenarios in a number of organisms including yeast and human.

**Results:**

Here we report on a new version of the tool, ANAT 2.0, which introduces substantial code and database updates as well as several new network reconstruction algorithms that greatly extend the applicability of the tool to biological data sets.

**Conclusions:**

ANAT 2.0 is an up-to-date network reconstruction tool that addresses several reconstruction challenges across multiple species.

## Background

The ANAT (Advanced Network Analysis Tool) [1] tool infers functional networks of proteins that underlie molecular processes in the cell. It is publicly available as a Cytoscape plug-in, providing a user friendly query interface, visualization of results, and network evaluation reports. On the server side, ANAT utilizes molecular interactions from several sources that were integrated and further refined into a reliable database for human, fly, yeast and rockcress. Building on this database, ANAT offers two types of network inference: approximate and optimal, with rigorous quality guarantees and highly efficient performance [2]. The output networks are visualized and statistically analyzed in various ways.

The first step in using ANAT is defining a set of *terminals* – proteins of interest, often obtained by observing the change in gene expression as a result of a process of interest. Next, the user submits a query by setting terminals, parameters and a background network, which can contain multiple types of interactions such as protein-protein and protein-DNA ones. ANAT supports four types of queries, resulting in different outputs: (i) “Anchored”, corresponding to the case where the process driver proteins – *anchors* – are known. In this case ANAT aims to infer a parsimonious network connecting anchors to terminals. (ii) “General” when no anchors are known. In this case ANAT infers a parsimonious network connecting the terminals. (iii) “Shortest paths” for inferring the most likely paths connecting pairs of anchors and terminals. And (iv) “Local search” for viewing the local protein neighborhood of the given terminals.

The network model inferred by ANAT is accompanied by a statistical report, including the chance probability of each node to be included in the model as well as the likelihood of each anchor-to-terminal path. The resulting model can be refined by incorporating expert knowledge (manually adding missing nodes or interactions, and excluding irrelevant ones). This allows for iterative cycles of analysis and refinement, striving to identify an accurate model.

In this paper we introduce ANAT 2.0, a comprehensive update of ANAT which includes the following new features: (i) an updated client side plug-in compatible with Cytoscape 3.5 and a new Java-based server which runs under Apache Tomcat; (ii) up to date protein-protein interaction (PPI) networks generated from the latest physical interaction datasets from BioGRID [3], with confidence scores estimated using a logistic regression-based scheme [4]; (iii) an implementation of the iPoint algorithm [5] for exact anchored network reconstruction; (iv) inference of end-point (terminal and anchor) sets for network reconstruction. For input sets that are derived from differential expression information, ANAT 2.0 allows inferring the transcription factors that govern the process under study, and uses them as terminals in the subsequent network inference. For data sets in which a natural anchor is not known, ANAT 2.0 provides the option to automatically generate an anchor set using a network propagation-based technique [6].

## Implementation

Implemented in Java, ANAT runs in Cytoscape 3.5 and newer versions. ANAT 2.0 is freely available at http://www.cs.tau.ac.il/~bnet/ANAT/, where the user can find installation instructions, documentation, user manual and sample inputs and outputs.

**Contact:** roded@tau.ac.il

## Results and discussion

**Fig. 1:**
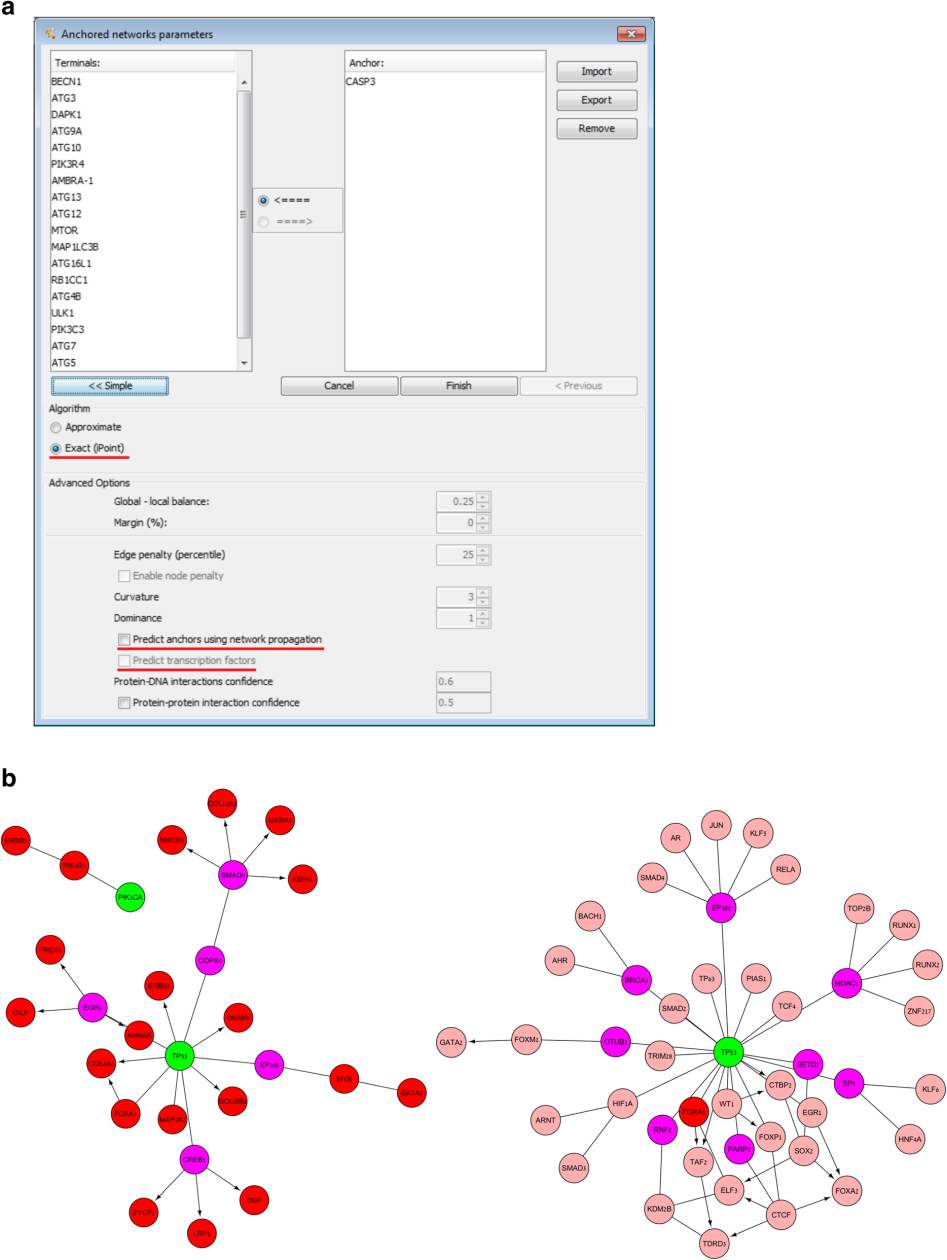
**a** The anchored network parameter dialog box. iPoint can be selected as an alternative “sub-algorithm” in the “Advanced” section, while anchor prediction and transcription factor prediction are available as additional options. **b** The network solution (left panel) and the network solution with “predict transcription factors” (right panel). The “predict transcription factors” reveals key transcription factors on a breast cancer dataset such as HIF1α and PARP1. The network shows the predicted pathways connecting the anchors (green) via connecting proteins (pink) and predicted transcription factors (salmon) to the terminals (red). Undirected edges correspond to PPIs and directed edges depict PDIs

Herein we describe the main new features of ANAT 2.0, depicted in Fig. 1a. In addition, the ANAT client has been rewritten to support the new Cytoscape 3.x API and repackaged as an Open Services Gateway initiative (OSGi) bundle. The server has been rewritten from the ground up as a standard Java-based web service which runs under Apache Tomcat. The construction and scoring of ANAT PPI networks (see Table 1) based on the BioGRID database has been standardized and automated. The network interactions are assigned confidence scores according to the experimental evidence that supports them, using the logistic regression framework described in Yosef et al. [4]. The training set for the logistic regression model consists of *positive* interactions from the KEGG PATHWAY database [7] and *negative* interactions chosen according to the distance between their end points (when the interaction is removed) following the work of Bader et al. [8]. In addition, regulatory protein-DNA interactions (PDIs) for the human and yeast networks were updated based on the Enrichr database [9] and YEASTRACT [10] (see Table 1).

**Table 1:** Numbers of nodes and edges in ANAT 2.0’s updated networks

Organism	Nodes	PPIs	PDIs
*H. sapiens*	20,933	251,078	271,314
*S. cerevisiae*	6352	75,620	150,843
*D. melanogaster*	8619	45,665	–
*A. thaliana*	9338	34,675	–

### Network reconstruction using iPoint

The ANAT network reconstruction engine aims to simultaneously optimize the size of the inferred network and the length of its anchor-to-terminal pathways. To this end it uses an efficient approximation algorithm. ANAT 2.0 offers in addition the iPoint exact subnetwork inference algorithm [5] as an alternative (see Fig. 1a). Unlike the approximation-based algorithm, iPoint requires no parameters for tuning, and returns an optimum solution by solving an elaborate integer linear program. For a range of practical cases, where the number of terminals is at most 20, the exact inference algorithm may be ideal. For other cases in which the execution time of iPoint exceeds 30 min, ANAT 2.0 prompts the user and advises to switch to the approximation-based algorithm (nonetheless providing the user also the option to run iPoint to completion).

### Pathway search from source to transcription product

A signaling pathway often targets a transcription factor, thereby affecting transcription. To support a subnetwork search that includes transcription factors as terminals we added the “predict transcription factors” feature (see Fig. 1a). This option searches for transcription factors that are significantly associated with the query terminals, by performing a hypergeometric test based on the enrichment of the query terminals within the set of known targets of a transcription factor (derived from its set of protein-DNA interactions). The algorithm computes a solution subnetwork connecting the query anchors to the identified transcription factors and their associated terminals. This solution refines the set of terminals by eliminating those that are not associated with an enriched transcription factor, potentially reducing the noise in expression measurement. It was shown useful in previous work [11], and we demonstrate its utility in the case study below.

### Case study

We demonstrate the utility of ANAT 2.0 and its new features by analyzing a breast cancer dataset. We downloaded breast cancer patient data from TCGA [12] and extracted the two most frequent genes (TP53 and PIK3CA) to serve as anchors and the 20 genes with highest mean value of differential expression to serve as terminals. We executed ANAT 2.0 using the human PPI and PDI network on these data both without and with predicting transcription factors. The second network solution refines the first (Fig. 1b), highlighting key transcription factors, which are significantly enriched with known cancer genes (58% included in the cancer gene lists from Hofree et al. [13], hypergeometric *p* = 5.3*е* ^−14^ only 5% included in the negative control list from [13], hypergeometric *p* = 0.94). Of particular interest is HIF1α, Hypoxia-inducible factor 1-alpha, a transcription factor known for its important role in promoting cancer through regulation of hypoxia processes, ultimately leading to increased angiogenesis and change in glucose metabolism which are fundamental for cancer growth [14]. This subnetwork also includes BRCA1 and PARP1, which are key to breast and ovarian cancer therapy [15].

### Anchor prediction using network propagation

For a data set in which a natural anchor is not known, ANAT 2.0 provides the option to automatically generate a suitable anchor set using a network propagation-based technique (Fig. 1). The terminal set is used as the basis for an initial propagation [6], with the terminals’ priors set to 1 and all others set to 0. The propagation process iteratively updates the score of a node as a linear combination of its prior score and the average scores of its neighbors until convergence. The top 100 scoring non-terminal proteins from this initial propagation are then considered as candidate anchors. To assign these candidates a *p*-value, ANAT 2.0 runs 100 additional propagations from random sets of the same size as the terminal set. Proteins that score better in the original run than in the random runs are retained. Up to 3 top scoring proteins that are not connected to each other are reported as the final anchor set.

## Conclusions

ANAT 2.0 provides multiple network inference solutions, suitable for a wide range of research needs: inferring anchored pathways from one or several focal points (given as input or predicted by the algorithm) to one or several affected proteins, and inferring regulatory pathways through a set of transcription factors that are associated with the given set of terminals. ANAT 2.0 interactive and easy to use interface enables its immediate integration in routine research procedures.

## Availability and requirements

Project name: ANAT 2.0.

Project home page: http://www.cs.tau.ac.il/~bnet/ANAT/

Operating system(s): Operates on all major operating systems.

Programming language: Java.

Other requirements: Cytoscape 3.5 and newer versions.

License: Free for academic use.

Any restrictions to use by non-academics: License needed.
